# Magnetic resonance image–guided focused ultrasound robotic system for transrectal prostate cancer therapy

**DOI:** 10.1002/rcs.2237

**Published:** 2021-03-03

**Authors:** Marinos Giannakou, Theocharis Drakos, Georgios Menikou, Nikolas Evripidou, Antria Filippou, Kyriakos Spanoudes, Leonidas Ioannou, Christakis Damianou

**Affiliations:** ^1^ R&D Medsonic LTD Limassol Cyprus; ^2^ Electrical Engineering Department Cyprus University of Technology Limassol Cyprus; ^3^ Radiology Department Ygia Polyclinic Limassol Cyprus

**Keywords:** focused ultrasound, MRgFUS, MRI, prostate, robotic device

## Abstract

**Background:**

A magnetic resonance image (MRI) guided robotic device for focussed ultrasound therapy of prostate cancer (PC) was developed. The device offers movement in 5 degrees of freedom (DOF) and uses a single‐element transducer that operates at 3.2 MHz, has a diameter of 25 mm and focuses at 45 mm.

**Methods:**

The MRI compatibility of the system was evaluated in a 1.5 T scanner. The ability of the transducer to create lesions was evaluated in laboratory and MRI settings, on ex vivo pork tissue and in vivo rabbit thigh tissue.

**Results:**

Cavitational and thermal lesions were created on the excised pork tissue. In vivo experiments proved the efficacy of the system in ablating muscle tissue without damaging intervening areas.

**Conclusions:**

The MRI compatible robotic system can be placed on the table of any commercial MRI scanner up to 7 T. The device has the ability of future use for transrectal focal therapy of PC with the patient in supine position.

## 1 INTRODUCTION

Prostate cancer (PC) is the second most common form of cancer malignancy presented in men after lung cancer, and the seventh cause of cancer‐related death worldwide.^1^ In 2018, it globally accounted for nearly 1.3 million newly registered cases and 360 thousand recorded mortalities.^2^

The 5‐year prevalence of PC in Europe is approximately 42% which is the highest rate among all cancer types.^1^ ^,^ ^2^ PC is more prevalent in males older than 55 years of age while it rarely affects those under 40 years old.^1^ An African‐American decency and a family history of PC increase its prevalence.^1^ The majority of age standardised incidence rates are presented in Australia with higher age standardised mortality rates recorded in Southern Africa.^1^ ^,^ ^2^

The implementation of prostate‐specific antigen (PSA) as a screening test for indication of PC presence, has led to increased number of registered cases at an early stage, often achieving a 10‐year timeframe from diagnosis to symptoms outbreak.^1^ PSA tests, Gleason scores and tumour stages, categorise patients into low, intermediate and high risk. Several treatment options are offered based on the advancement of the disease. These include active surveillance (AS), radical prostatectomy (RP), external beam radiation therapy (EBRT), interstitial brachytherapy and androgen deprivation treatment (ADT).^3^

Focal therapy including laser ablation, cryotherapy and high‐intensity focused ultrasound (HIFU) has been developed for low or intermediate risk patients so as to treat only the malignant tissue leaving the benign tissue (urethra, neurovascular bundles) unaffected.^4^ It offers an alternative middle‐point solution between whole gland radical treatment and AS. Laser ablation was introduced in 1990 for the treatment of benign prostate hyperplasia^5^ (BPH) and has since been used in several clinical trials for the treatment of PC with promising results.^6^ ^,^ ^7^

Cryoablation is a more frequently used technique, introduced in 1966 for the treatment of BPH,^8^ and later used for prostatic neoplasms^9^ although, with minor tumour ablation and severe complications. Advancements in the technology have since resulted in widespread use with high survival rates and low complication rates.^10^

HIFU exists as a technology since 1940 and uses high‐intensity ultrasound waves focused on a single point to cause high increases in tissue temperature. This causes cell necrosis thus disrupting their infinite neoplastic proliferation. Ultrasound (US) and MRI are used during treatments to provide anatomical image feedback.

Commercially, there are two U.S.‐guided FDA‐approved HIFU systems for PC treatment: Sonablate (Sonacare) and Ablatherm (Edap‐TMS). Ablatherm incorporates two individual transducers operating at 3 and 7.5 MHz used for therapy and imaging respectively,^11^ while Sonablate uses a single transducer operating at 4 MHz for both therapy and imaging.^12^ Additionally, differences in patient position, treatment protocols and planning exist between the two devices.^11^ ^,^ ^12^ The two devices have been extensively used in clinical trials to prove their efficacy, with a majority of them performed using the Ablatherm device. The first use of HIFU on malignant PC was done in 1992 on ablating Dunning R3327 adenocarcinoma on rats using an Ablatherm prototype. A complete ablation was achieved in 64 % of the cases, resulting in higher efficacy rates than other modalities reported in literature.^13^ Madersbacher et al.^14^ were the first to use the Sonablate device on 29 human prostates before RP, where they concluded that the ablation of 20 ml of prostatic tissue was safe without unwanted changes in the intervening rectal wall. While HIFU is not recommended for high‐risk patients, when combined with transurethral resection of the prostate (TURP) it can offer an alternative treatment^15^ ^.^

The adverse side effects induced by the two systems include fistula and urethral stenosis and incontinence, with the former rarely being reported^16^, ^17^, ^18^, ^19^ and the latter more commonly being indicated.^16^ Incontinence rates slightly increase after multiple HIFU treatments;^20^ however, significant improvements in bladder function are achieved 6 months posttreatment.^21^ When compared to brachytherapy, 5‐year cancer‐specific and survival rates do not differ between the two modalities, with HIFU achieving PSA nadir at a shorter time.^22^ Cancer specific survival rates after HIFU treatment of 100% and 98% at 5‐year and 8‐year respectively have been noted.^23^

The use of MRI as a guidance modality optimises the efficacy of tissue ablation by providing real‐time feedback of in situ temperature increase as well as providing higher tissue contrast. Robotic systems used inside MRI environments need to be constructed of materials that do not result in hazardous projectiles due to the high magnetic field, do not interfere with image acquisition nor is their functionality affected by the strong electromagnets. The majority of robotic systems are actuated by hydraulic, piezoelectric or pneumatic motors. Piezoelectric motors are advantageous due to their small size however, their motion is obstructed during imaging, contrary to pneumatic motors which are the most MRI compatible with no loss in signal to noise ratio (SNR).^24^ Nevertheless, the source of pressure required for hydraulic and pneumatic motors demands large and complex robotic systems.

The first MRI guided HIFU robotic system was patented by General Electric in 1993 and was developed with hydraulic actuators.^25^ Thenceforth, there has been a gradual evolution of MRI compatible HIFU robotic systems for a variety of applications.^26^ The majority of those systems utilize piezoelectric actuators, firstly introduced by the Israeli company Insightec^27^ to compensate for accuracy and MRI interference problems induced by hydraulic motors. Robotic systems for MRI‐guided focussed ultrasound (MRgFUS) treatment of PC were motivated by MRI compatible systems for other prostate interventions such as biopsy and brachytherapy. The majority of the systems offer motion in more than 3 degrees of freedom (DOF), with some of them employing piezoelectric actuators for both biopsy and brachytherapy^28^ ^,^ ^29^ or pneumatic actuators for sole brachytherapy use.^30^ The widespread use of piezoelectric motors has led to the recent development of 6 DOF robotic system for both biopsy and brachytherapy as well as laser ablation of the prostate.^31^ However, the aforementioned systems cannot be used for focused therapy since they do not provide the spatial requirements for addition of water coupling necessary for ultrasound propagation.

There are two novel Conformité Européenne (CE)‐marked MR‐guided ultrasound systems for the treatment of PC. The ExAblate (Insightec) system^32^ offers a transrectal probe with 990 phased array focused elements for PC ablation while the TULSA‐PRO (Profound Medical) offers a transurethral probe with a linear array of 10 single unfocused elements.^33^ The ExAblate system was first evaluated in 2009 in the preclinical setting on canine prostate model,^34^ while the first clinical experience on 5 patients with PC before RP was performed in 2012.^32^ While no thermal necrosis was observed in the preclinical study due to limitations on dog size, histopathology specimens revealed complete necrosis at the sonication site in the clinical study. Further studies confirmed device efficacy, with minor complications.^35^ ^,^ ^36^ Clinical trials have also been performed with the TULSA‐PRO achieving low PSA levels but with increased rates of haematuria and urinary tract infections^33^ due to the transurethral approach to treatment.

The developed robotic system is guided by MRI thus achieving near real‐time temperature monitoring of the ultrasonic exposure. It includes three linear stages and two angular stages thus offering movement of the transrectal focused ultrasound (FUS) transducer in 5 DOF. This system is an improvement compared to our previous designs,^37^, ^38^, ^39^ offering improved aesthetics and better ergonomics of the device.

## 2 MATERIALS AND METHODS

### 2.1 Robotic system

- Mechanical design of the positioning device

The robotic system was designed using the software MicroStation (V8, Bentley Systems, Inc.) and printed using an industrial 3D printer (FDM 270, Stratasys) that produces parts from Acrylonitrile Butadiene Styrene (ABS).

The robotic device has been developed to offer movement in 5 axes (4 computer controlled and 1 manual). The four computer controlled axes include a linear axis for motion of the transducer within the probe and along the rectum (X), an angular axis for rotation of the transducer within the rectum (Θ), a linear axis for height adjustment (Z) since there is variability of rectum height between patients and an angular axis (Φ) that adjusts the entry angle to the rectum. The manually controlled linear axis (Y) provides left to right movement on the base of the device. Motion in each of the axes is achieved through piezoelectric motors (USR60‐S3N, Shinsei Kogyo Corp.) combined with optical encoders (EM1‐0‐500‐I, US Digital Corporation) for accurate movement.

**FIGURE 1:**
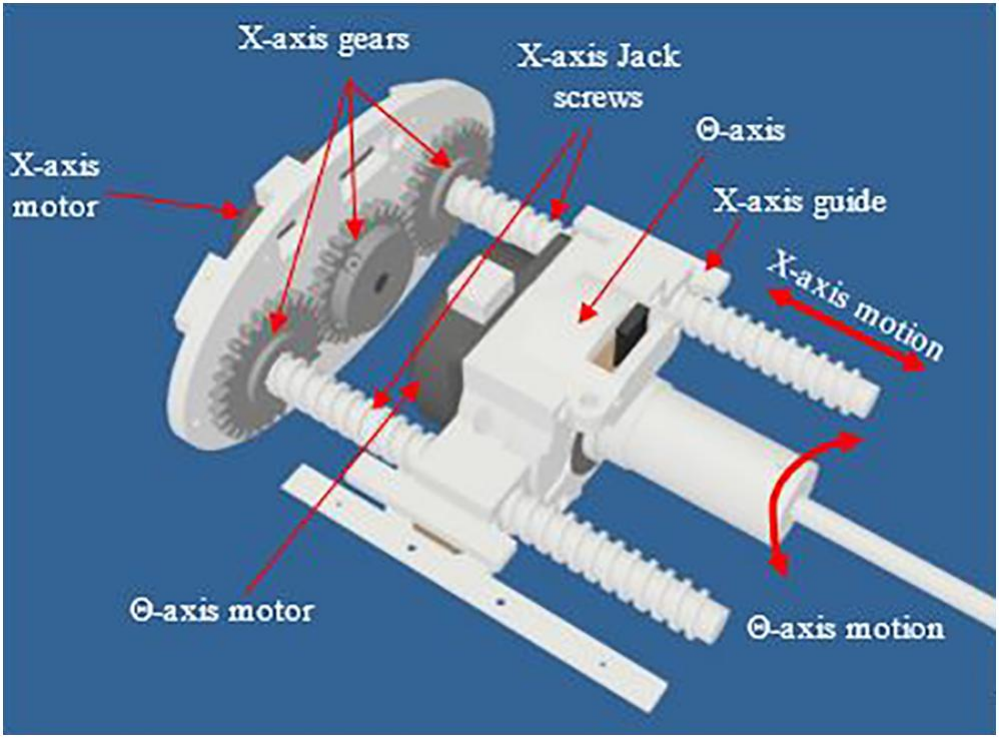
CAD drawing of the linear X axis. CAD, computer‐aided design

The X axis has a motion range of 50 mm. Figure 1 shows the computer‐aided design (CAD) of the X axis. Motion is achieved through a pinion gear attached to the ultrasonic motor through a motor holder. The pinion gear was coupled with two spur gears each attached to a jackscrew mechanism. The X axis jackscrews were coupled to the Θ axis motor holder. The use of two jackscrews enables the application of uniform force on both sides of the Θ axis.

**FIGURE 2:**
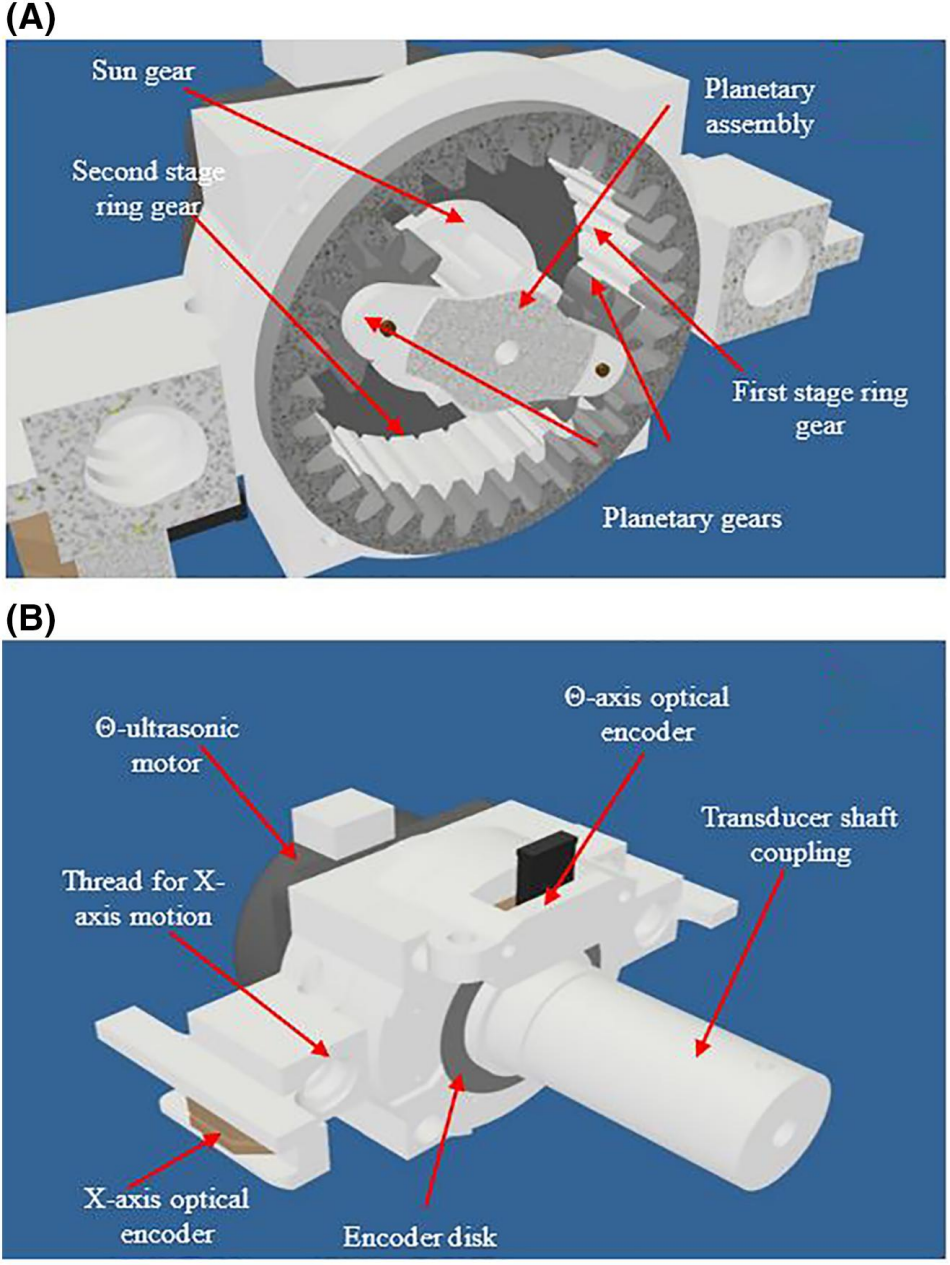
(A) Cross section of the Θ axis motion mechanism. (B) CAD design of the complete angular Θ axis. CAD, computer‐aided design

**FIGURE 3:**
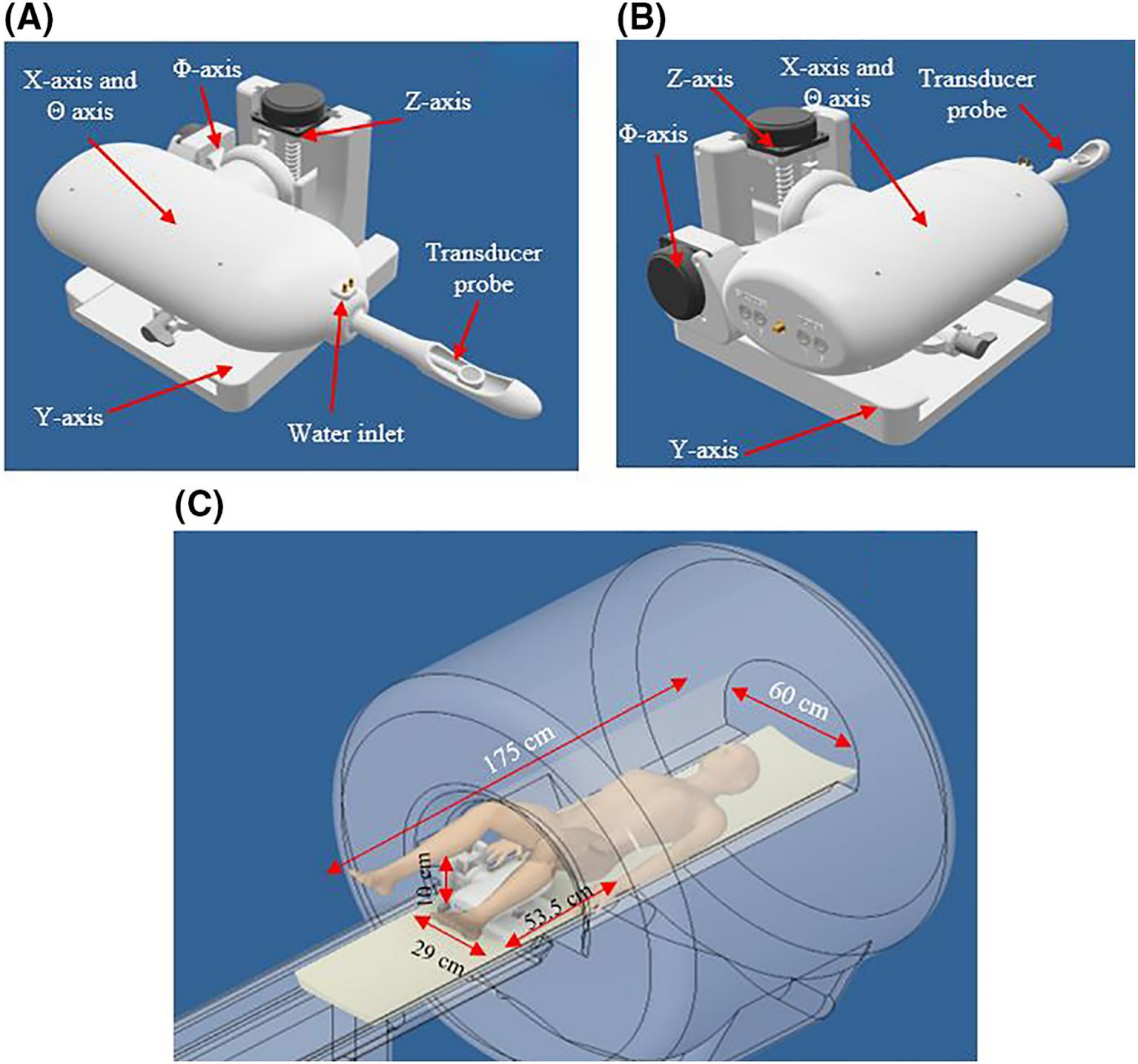
(A) Front view of the CAD design of the robotic system. (B) Rear view of the CAD design of the robotic system. (C) Introduction of the device as placed on the MRI table. CAD, computer‐aided design; MRI, magnetic resonance image

The Θ axis was the most complex mechanism of the device since it uses a two‐stage planetary gear mechanism. Figure 2A shows the cross section of the motion mechanism of the axis whereas Figure 2B shows the CAD design of the complete Θ axis. A sun gear is coupled to the motor and placed at the centre of the mechanism. Two planetary gears are rotated by the sun gear inside the first stage ring gear. The planetary gears are simultaneously coupled to the second stage ring gear which is designed with one tooth less than the first gear. This results in smooth rotation of the second stage and therefore rotation of the coupled transducer shaft in a ±90° range. Z axis uses a jackscrew mounted on the motor for the linear motion of the device on a vertical direction. Movement in the Φ axis was achieved through a worm gear, attached to the Φ axis motor. A worm gear was coupled to a ring gear thus adjusting the angle of the robotic device. Figure 3A shows the front view of the complete CAD design of the device. Figure 3B shows the rear view whereas Figure 3C shows the configuration and placement of the device inside the MRI scanner. The patient is lying on supine position on the table with his legs slightly elevated to provide rectum access for the transducer probe.

The dimensions of the device are 53.5 cm in length, 10 cm in height, 18 cm in width with a 3 cm wide probe, on a 25 × 29 cm base. The device weighs 3 kg and can be placed on the table of magnetic resonance (MR) scanners up to 7 T.

The transrectal probe is fitted to a condom filled with degassed water hence providing proper ultrasound propagation. The degassed water was circulated during evaluation through a water inlet attached to the probe (Figure 3A). A custom‐made cooling system consisting of two high‐flow water pumps (AD20P‐1230C, Giant Electric Tech. Inc.), a high‐pressure pump and 10 Peltier modules (TEC1‐12706), was used for water circulation into the probe, thereby achieving transducer cooling.

b. Software

The computer‐controlled axes of the robotic system can be controlled with the aid of a software developed with C # (Visual Studio 2010 Express, Microsoft Corporation). The software provides choice of manual or automatic movement of the controlled axes as well as control of the sonication parameters (frequency, power, sonication time, etc.). Moreover, it has the ability to store patient information, communicate with the MRI and provide MR thermometry maps.

c. Electronic system

The rotation of each ultrasonic motor is controlled by a driver (D6030, Shinsei Kogyo, Corp.) that operates using a DC power supply (24V, 6A). The drivers are enclosed within a metallic housing that was placed outside the MRI room. The movement is controlled through a data acquisition interface card (DAQ, USB 6251, National Instruments).

### 2.2 HIFU system

The system consists of a RF generator (AG Series Amplifier, model AG1012, T&C Power Conversion Inc.) that operates a custom‐made spherical transducer (MEDSONIC LTD, Limassol, Cyprus). A two component‐epoxy (ASonic) was chosen as a backing material, preventing excessive vibrations of the piezoceramic element (Piezo Hannas Tech Co.). The transducer operates at 3.2 MHz, has a focal length of 45 mm and a diameter of 25 mm.

### 2.3 MR compatibility

The transducer and robotic device were individually evaluated for MR compatibility in a 1.5 T MR scanner (Signa, General Electric) using a GPFLEX coil (USA Instruments). An agar‐based phantom containing 6 % w/v agar (10164, Merck KGaA) and 30 % v/v evaporated milk (Nounou, Friesland Campina) and an MR cylindrical phantom (containing nickel sodium solution (3.3685 g/L NiCl; 6H2O; and 2.4 g/L NaCl), USA Instruments) were utilised for evaluation of the compatibility of the transducer and robotic device respectively.

The MR compatibility was evaluated by measuring the SNR utilising a method in the National Electrical Manufacturers Association (NEMA) standard.^40^ Two images of the phantom (agar or MR) are acquired under the same conditions. The SNR was calculated by dividing the signal of the first image (S_image1_) with the standard deviation of the pixel‐by‐pixel difference of the two images (SD|_image1‐image2_|) as shown in the following Equation (1):

(1) $$SNR=\surd 2\;\frac{S_{\text{image}1}}{SD{|\text{image}1‐\text{image}2}_{\;}|}$$

The compatibility of the transducer and robotic device was evaluated using T2 Weighted‐Fast Relaxation Fast Spin Echo (T2‐W FRFSE), Fast Spoiled Gradient (FSPGR) and Echo Planar Image (EPI) sequences. These sequences were selected since the first is used for high‐resolution imaging while the other two for extraction of MR thermal maps. Regarding the transducer, the baseline image (S_image1_) was taken with only the agar phantom in the MR bore. Images were then acquired for both amplifier and transducer deactivated, for activated amplifier and deactivated transducer and for both amplifier and transducer activated. Hence, the standard deviation of the difference between the baseline, and images taken in each condition was found, and thus SNR was calculated. For the T2‐W FRFSE sequence, activation of the transducer was not followed, since this sequence is not intended for MR thermometry.

Concerning the robotic device, the baseline image was taken with only the MR phantom in the bore. Images were then obtained for the robotic device in the bore (no cables attached), the robotic device wired but with deactivated electronic system, and the connected device with powered electronic system. The SNR was calculated for each of the aforementioned conditions.

Given that all components require the use of electricity for activation, the device is classified as MRI‐conditional according to the American Society for Testing and Materials (ASTM) standards (F2503, F2052, F2213, F2182, F2119).

### 2.4 Laboratory and MRI ex vivo evaluation of the FUS system

The performance of the transducer was evaluated in the laboratory and in MRI setting on freshly excised porcine loin tissue. The tissue was purchased before conduction of the experiment and was chosen to be homogeneous (no fat present).—Initially, the focal point of the transducer within the ex vivo tissue was located by depositing the transducer in a holder facing the tissue surface. A thermocouple (HH806AU, Omega Engineering) was inserted inside the target tissue at set distances of 5 mm whilst sonication at constant acoustical power of 15 W for 30 s was performed at each distance. Thereby, temperature measurements taken at various depths, resulted in near‐field and far‐field temperature profiles of the ultrasonic beam within the target.

After locating the focus, higher acoustical power was used for the creation of discrete lesions in a grid pattern. The experiment was performed in the laboratory setting at constant acoustical power of 60 W for 6 s for the formation of lesions in a 3 × 3 pattern. The experiment was also implemented in the MRI environment using varied sonication parameters. Sonication was performed in a 3 × 2 pattern at focal depths of 2 and 3 cm, acoustical powers of 43 and 57 W and varied sonication times. After conduction of the experiments, the excised tissue was sliced, and the dimensions of the lesions were measured using a digital calliper (ROHS NORM 2002/95/EC).

### 2.5 MR thermometry

MR thermometry data were produced using the proton resonance frequency shift method^41^ which relates the measured phase shift with the change of temperature (Δ*T*). This relationship is given by:

(2) $$\Delta Τ\;=\;\frac{\varphi \left(Τ\right)\;-\;\varphi \left({Τ}_{0}\right)}{\gamma \alpha {Β}_{0}ΤΕ}$$

where φ(*T*) and φ(*T*_0_) are the phases at starting and final temperature *T* and *T*_0_ respectively, γ is the gyromagnetic ratio, α is the proton resonance frequency shift coefficient (0.01 ppm/^ ^C), B_0_ is the magnetic field strength and TE is the echo time. A single shot EPI sequence was used for acquiring the MR thermometry maps. The sequence had a 1 s temporal resolution with the following parameters: Repetition Time (TR) = 80 ms, Time Echo (TE) = 25.5 ms, Field of View (FOV) = 15 cm, matrix = 64 × 64, flip angle = 25°, Number of excitations (NEX) = 4, Echo Training Length (ETL) = 1, delay = 1000 ms, slice thickness = 2 mm.

### 2.6 In vivo evaluation of the FUS system

**FIGURE 4:**
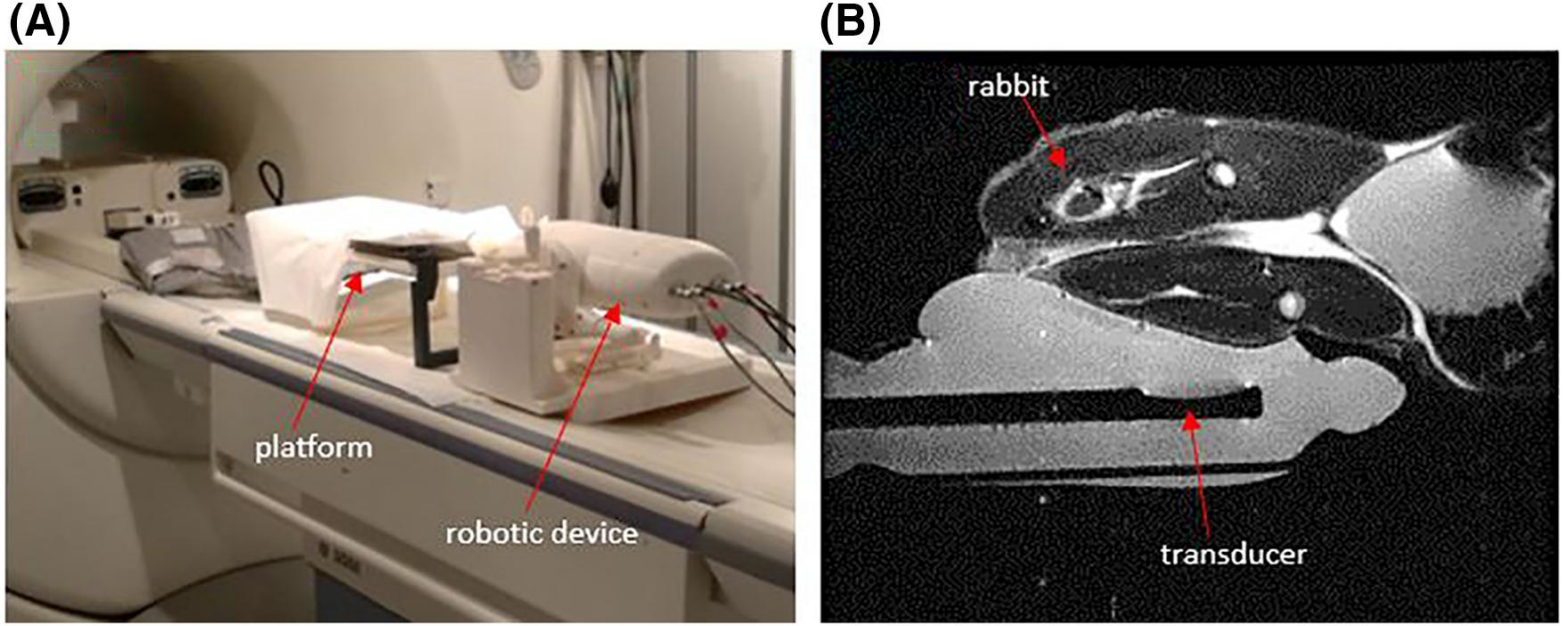
(A) Robotic device placement on MRI table. (B) High‐resolution T2‐W FRFSE image (sagittal plane) of the rabbit thigh and robotic device. FRFSE, Fast Relaxation Fast Spin Echo; MRI, magnetic resonance image

The robotic device was evaluated in the MRI setting for assessing the efficacy of the transducer in sonicating healthy thigh tissue on a rabbit model (*n* = 8) and determinating any serious side effects. The rabbits were purchased from an accredited farm and all experiments were performed under the approval of the Veterinary Services, Ministry of Agriculture, Cyprus. Prior to conduction of the experiments, the rabbits were anaesthetised with injectable Ketamine (0.15 mg/kg) and Medetomidine (0.5 mg/kg), and their thighs were depilated (VEET, Reckitt Benckiser). The robotic device was placed on the table of a 1.5 T MRI scanner (Signa, General Electric) and an ABS platform featuring an opening, was placed on top of the transrectal probe. The rabbits were then placed on the platform with their thigh situated on the opening thus allowing proximal contact with the probe which was covered with a condom (DUREX, Reckitt Benckiser) containing degassed water. A high‐resolution T2‐W FRFSE image of the set‐up was acquired to ensure good coupling between the thigh and the transducer probe. Additionally, the image was used for calculation of the available thigh area thus avoiding ultrasonic exposure of the bone. Figure 4A shows the placement of both the robotic device and platform on the MRI table, while Figure 4B shows the acquired T2‐W FRFSE image of the set‐up. Different sonication parameters (acoustic power, sonication time) were used to examine the effect of induced thermal dose on tissue necrosis. MR thermometry maps were acquired during sonications to evaluate the in situ temperature change. Proton density (PD) images with fat suppression were acquired after sonications so as to examine the ablated area. Vital signs (heart rate, respiratory rate) and urination were monitored during the experiment. Upon completion of the sonications, the rabbits were humanely euthanized with intracardial injection of T‐61 (MSD Animal Health) and ante mortem tissue dissection was performed to examine the extent of necrosis.

## 3 RESULTS

### 3.1 MR compatibility

**FIGURE 5:**
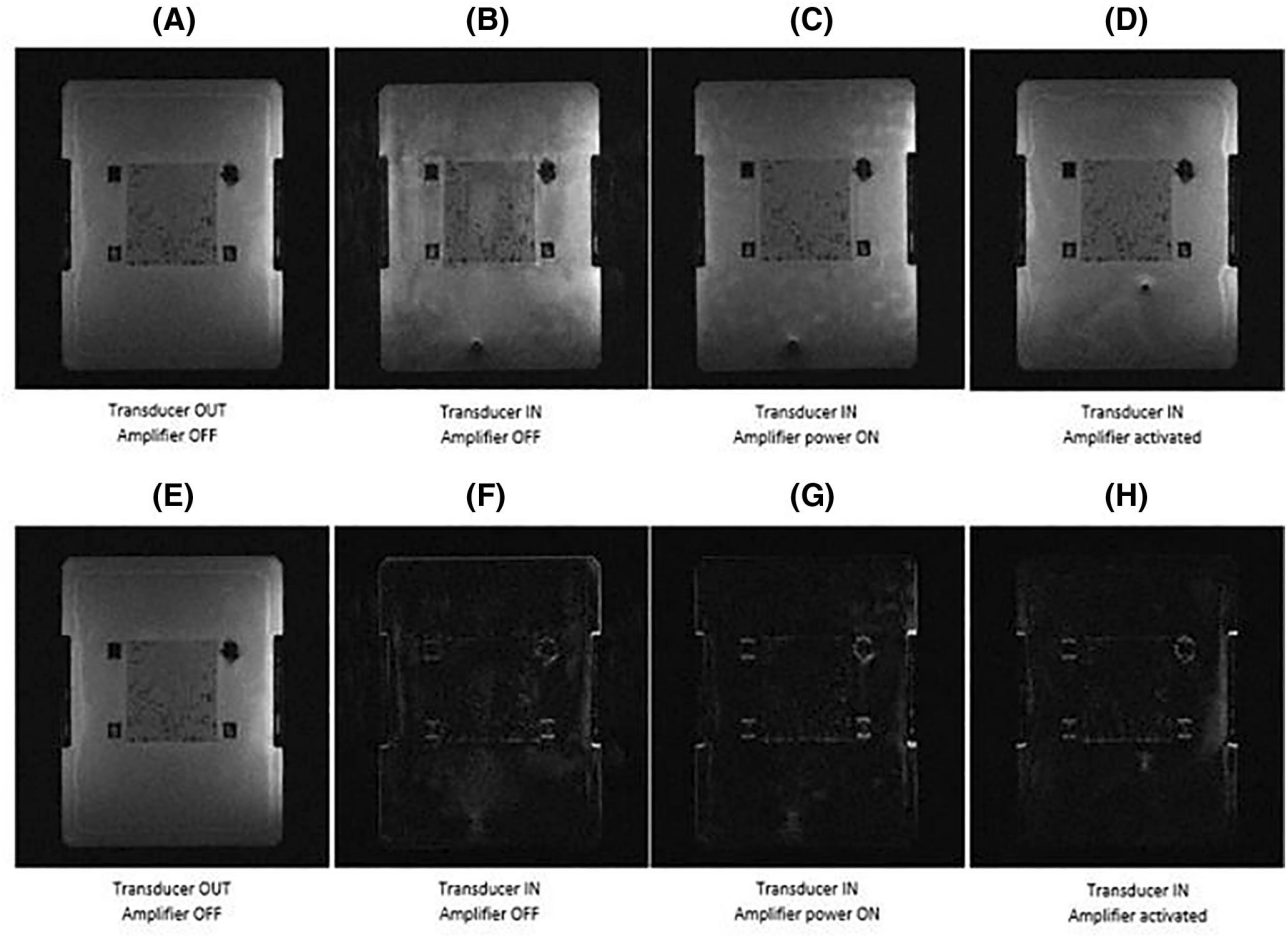
MR images (A–D) obtained using FSPGR for different activation conditions of the transducer and their corresponding differences (E–H) from the baseline image. FRFSE, Fast Relaxation Fast Spin Echo; MR, magnetic resonance

**FIGURE 6:**
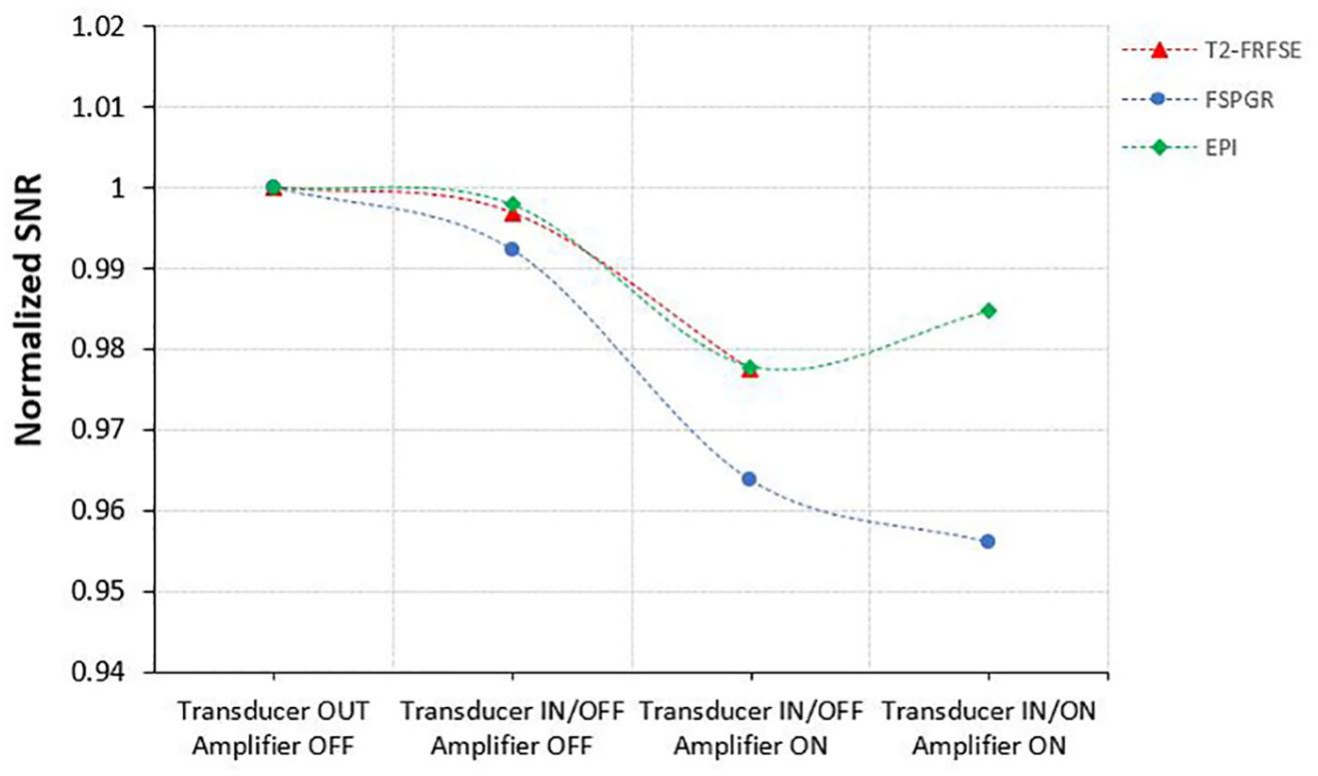
SNR using three MR imaging protocols (T2‐W FRFSE, FSPGR, and EPI) for different activation conditions of the transducer. EPI, Echo Planar Image; FRFSE, Fast Relaxation Fast Spin Echo; FSPGR, Fast Spoiled Gradient; MR, magnetic resonance; SNR, signal to noise ratio

**FIGURE 7:**
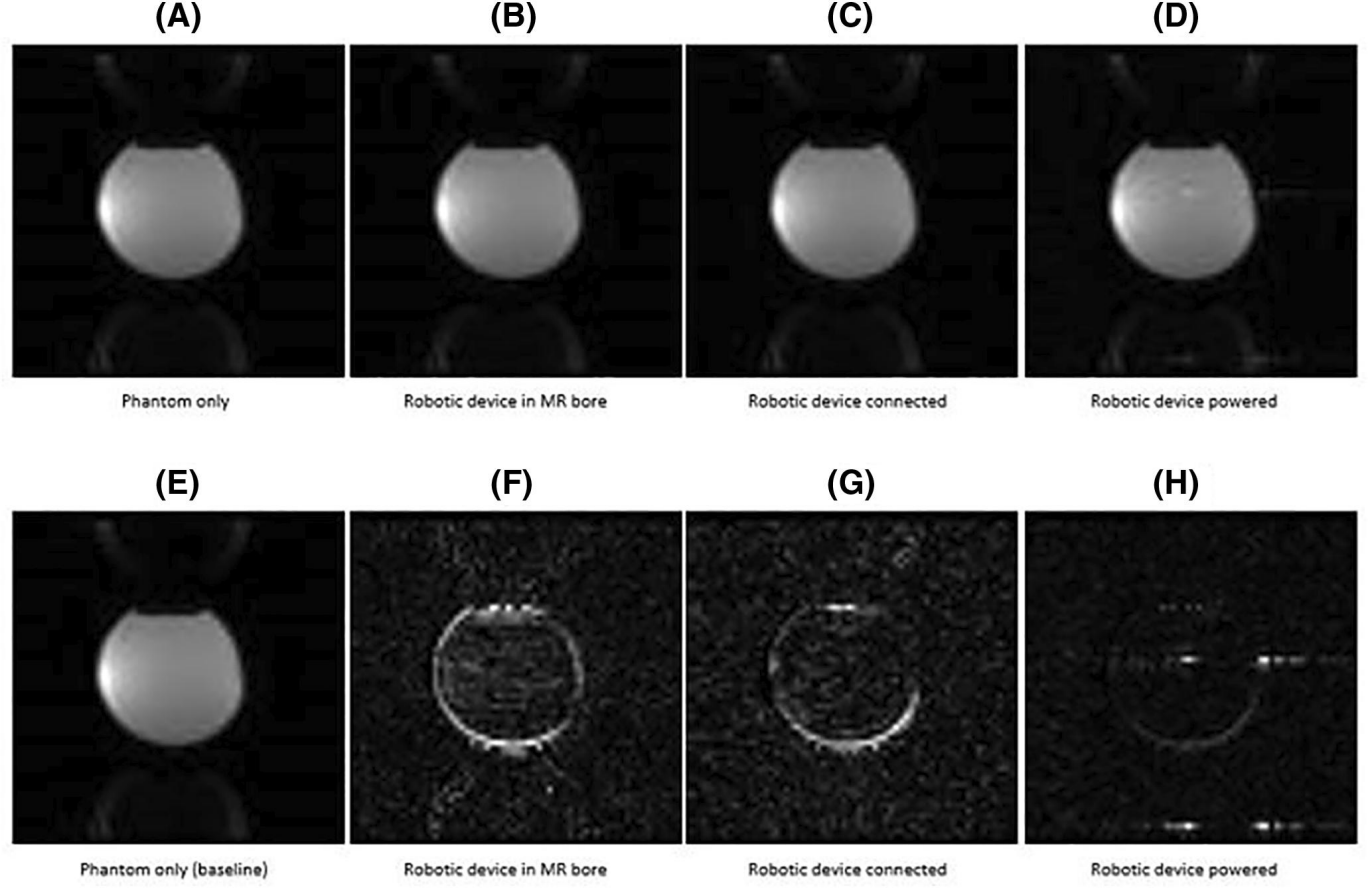
MR images (A–D) obtained using EPI sequence for different activation conditions of the robotic device and their corresponding differences (E–H) from the baseline image. EPI, Echo Planar Image; MR, magnetic resonance

**FIGURE 8:**
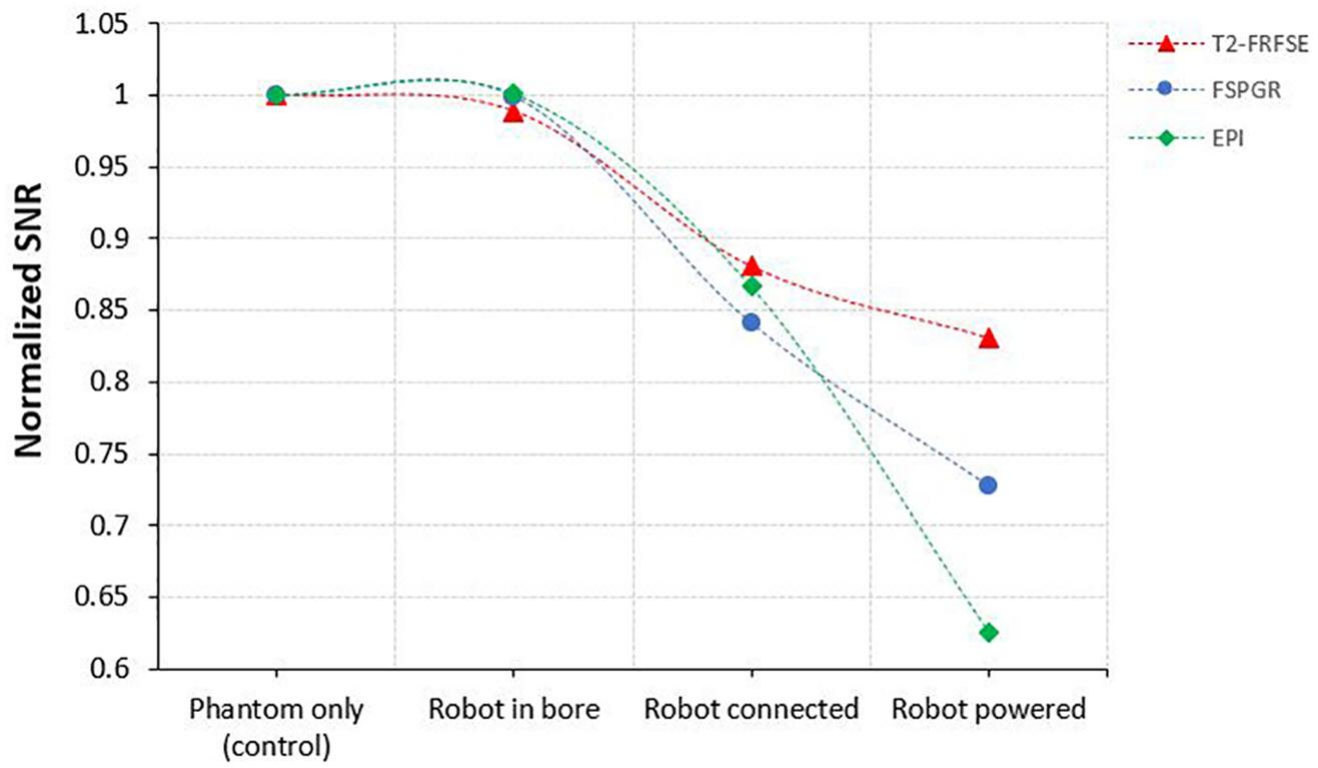
SNR using three MR imaging protocols (T2‐W FRFSE, FPSGR, and EPI) for different configurations of the robotic system. EPI, Echo Planar Image; FRFSE, Fast Relaxation Fast Spin Echo; FSPGR, Fast Spoiled Gradient; MR, magnetic resonance; SNR, signal to noise ratio

Figure 5 shows the MR images (top) acquired on coronal plane for different activation conditions of the transducer using FSPGR sequence and their corresponding differences (bottom) from the baseline image. Figure 6 shows the SNR as calculated from the MR images of the T2‐W FRFSE, FSPGR and EPI for the different activation conditions of the transducer. The SNR for different configurations of the transducer was slightly decreased upon connection and activation of the amplifier and did not significantly vary between T2‐W FRFSE and EPI. FSPGR resulted in the greatest reduction of SNR upon powering of the transducer. The SNR using EPI increased slightly (1%) upon activation of the transducer. It is possible that the change in the signal with EPI during activation of the transducer was so small that falls within the noise of the scanner, that's why the SNR appeared increased. Although the activation of the amplifier and transducer affects slightly the SNR, it did not significantly disturb the MR thermometry sequences. Figure 7 shows the MR images acquired using EPI sequence for different configurations of the robotic device while Figure 8 shows the SNR as calculated from the MR images of the T2‐W FRFSE, FSPGR and EPI. The SNR was not affected upon introduction of the robotic device in the bore, for all three sequences. There was an SNR reduction upon connection of the robot, with FSPGR resulting in the greatest decrease. On the other hand, EPI resulted in the largest SNR reduction during activation of the electronic system.

### 3.2 Focus location

**FIGURE 9:**
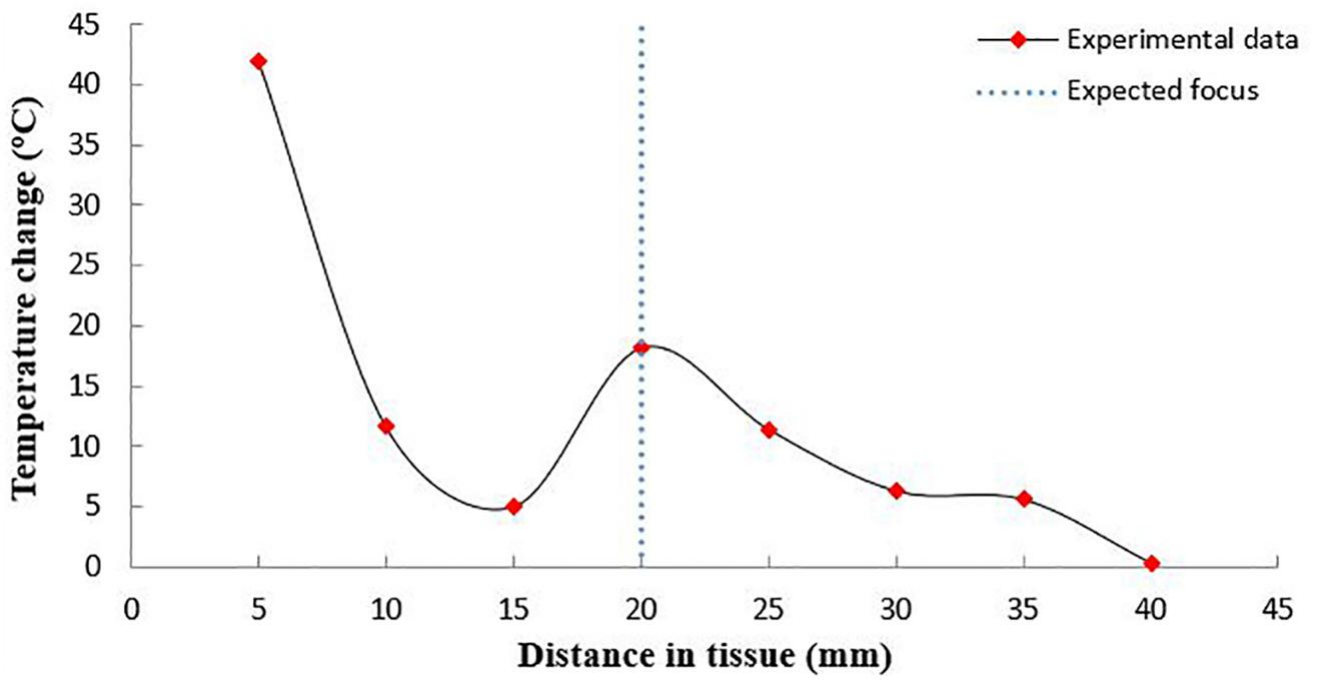
Temperature change versus depth in the porcine ex vivo tissue using acoustical power of 15 W for sonication time of 30 s for the transducer with frequency 3.2 MHz

**FIGURE 10:**
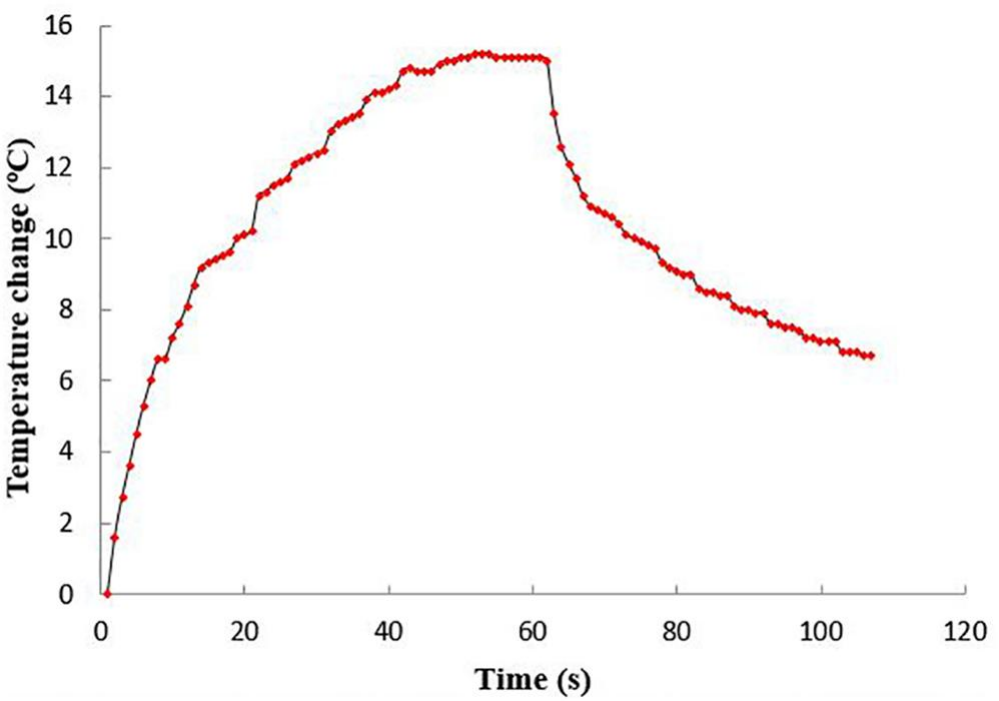
Temperature change versus time at the ex vivo porcine tissue focus for the transducer with frequency 3.2 MHz at acoustic power of 30 W for a sonication time of 60 s at 2 cm focal depth

**FIGURE 11:**
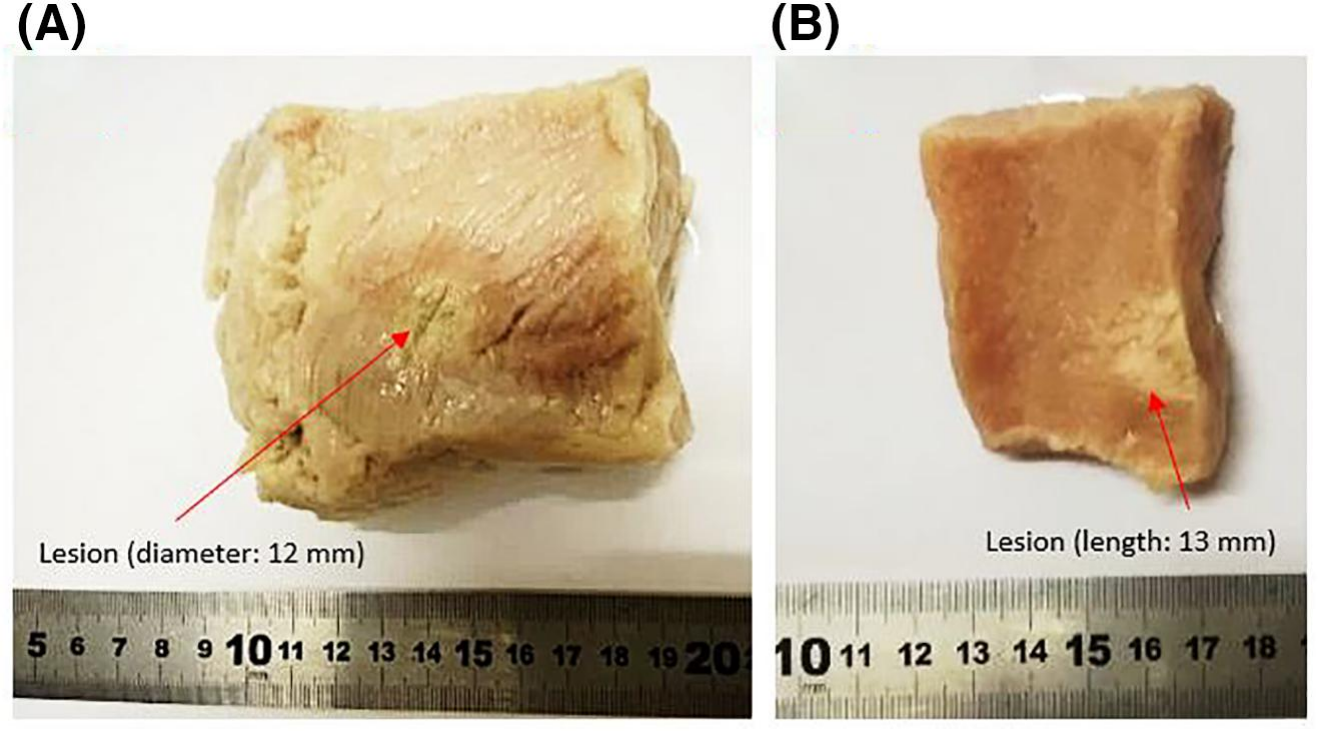
(A) Lesion formed on the ex vivo porcine tissue interface at a plane perpendicular to the beam. (B) Lesion formed at a plane parallel to the beam on ex vivo porcine tissue resulting exposure at 30 W, sonication time 60 s, for the transducer with frequency 3.2 MHz at 2 cm focal depth

Figure 9 shows the thermocouple‐measured temperature change versus the depth in the ex vivo tissue. The expected focus of the transducer was at 20 mm. However, there was a large increase of temperature recorded at the tissue interface (5 mm distance). Sonication was then performed at an acoustical power of 30 W for a sonication time of 60 s. Figure 10 shows the rate of change of temperature at the focus. This sonication created a lesion at the interface. Figure 11 shows the 12 mm wide and 13 mm long lesion formed at the interface.

### 3.3 Creation of discrete lesions on ex vivo tissue

**FIGURE 12:**
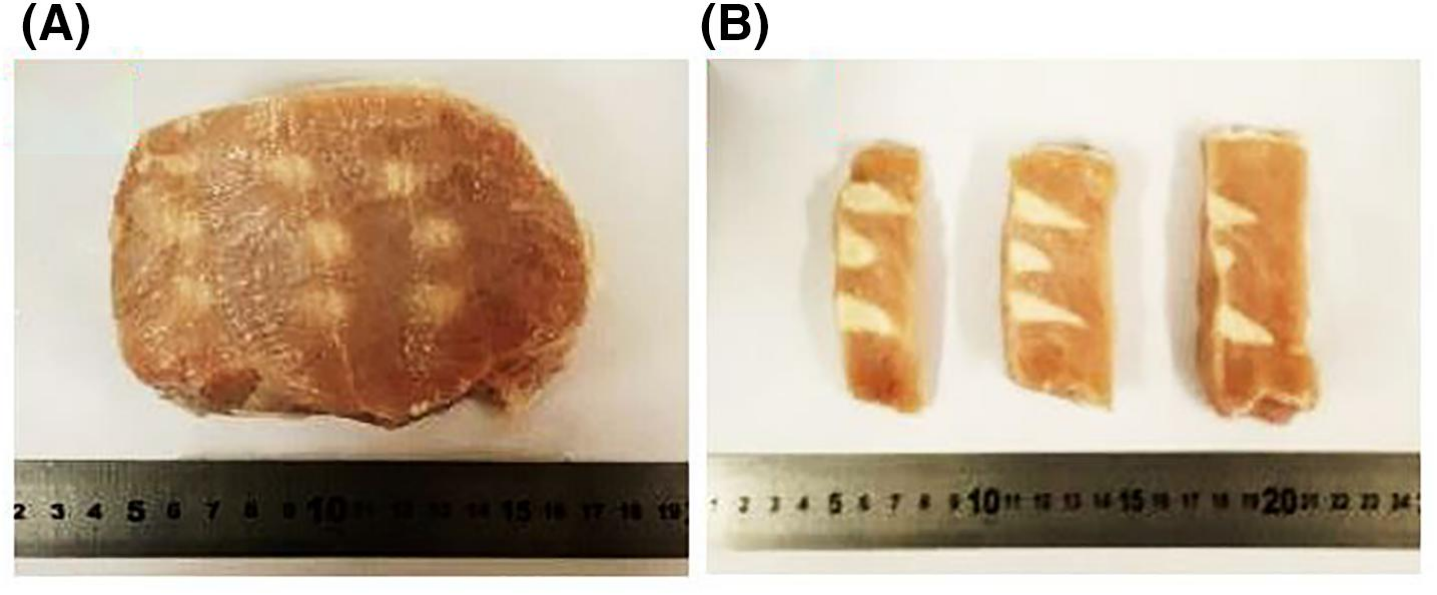
(A) Lesions formed in the pork tissue in a plane perpendicular to the beam. (B) Lesions formed in a plane parallel to the beam due to an exposure at acoustical power 60 W, sonication time 6 s, for the transducer with frequency 3.2 MHz at 3 cm focal depth

Lesions on ex vivo porcine were created in a laboratory environment using movement of the transducer. Figure 12 shows the excellent repeatability of the transducer. The formed lesions had an excellent repeatability in their diameter with a slight variation of length, indicating possible presence of air bubbles. This is also indicated from the tadpole shape of the lesions, concluding the presence of cavitation during lesion formation. The average diameter of the formed lesions was 8.2 mm and the average length was 17.3 mm.

**FIGURE 13:**
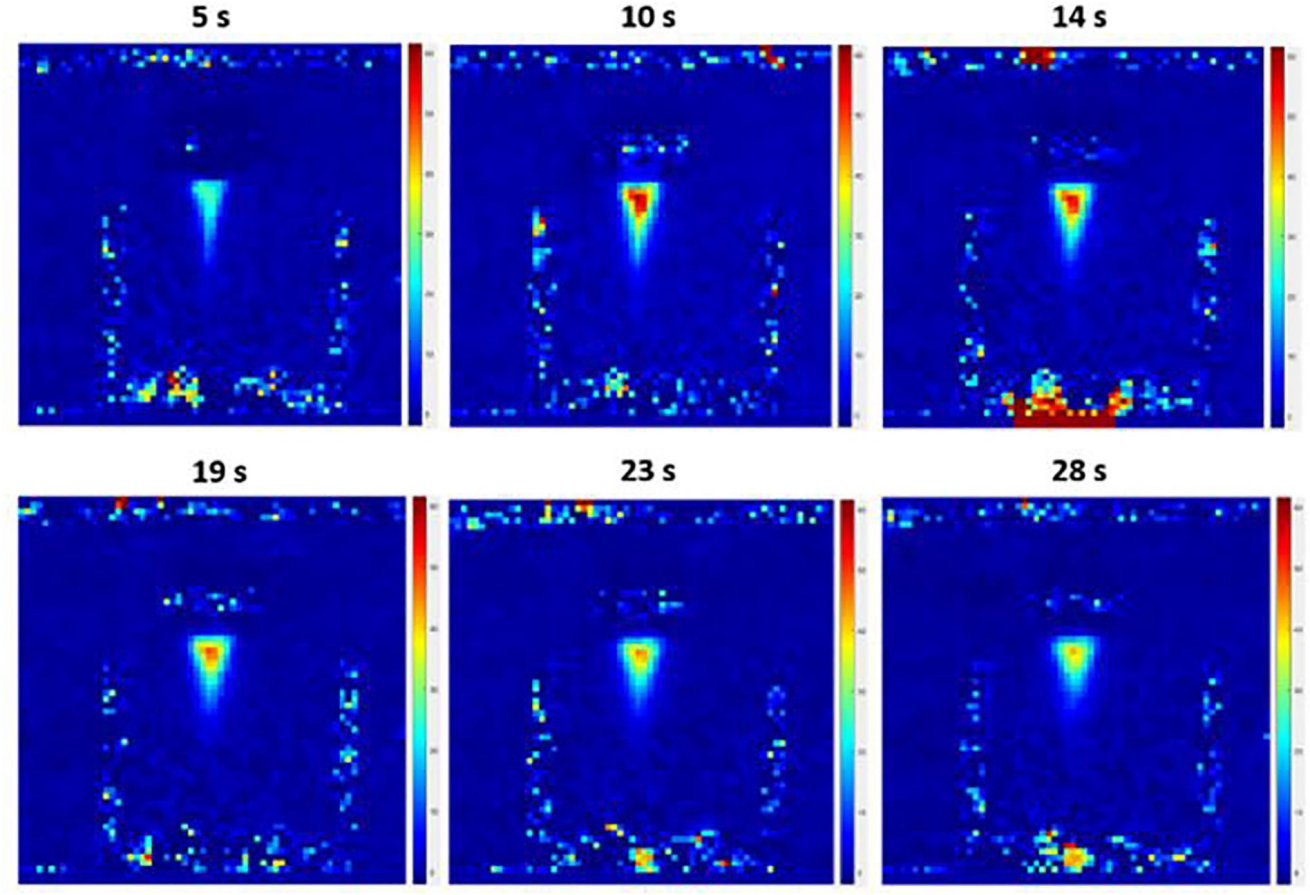
Temperature maps acquired on axial plane using EPI sequence at acoustical power of 57 W for sonication time of 10 s and cooling off time of 18 s. EPI, EPI, Echo Planar Image

**FIGURE 14:**
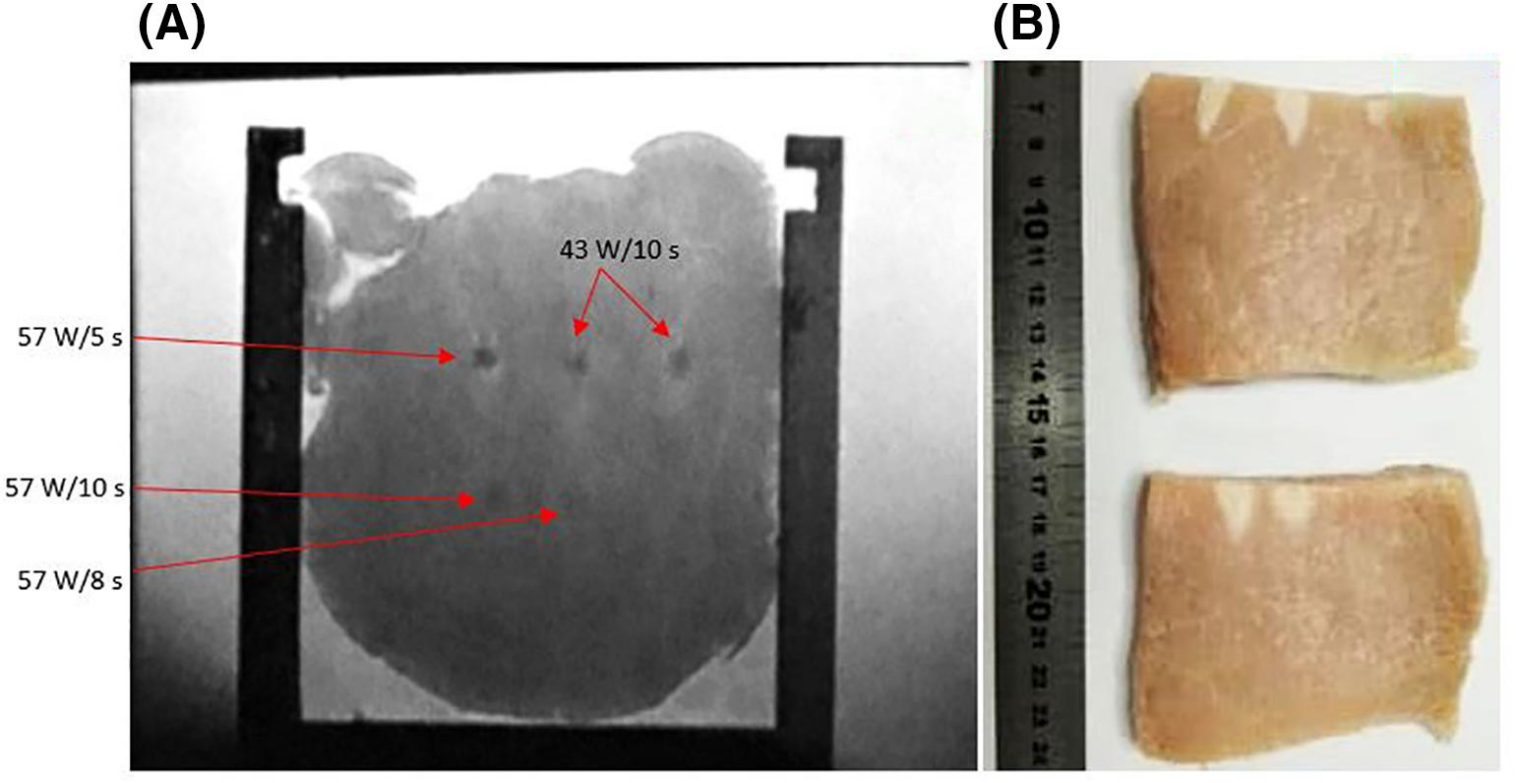
(A) High‐resolution PD with fat suppression MR image (coronal plane) obtained at plane perpendicular to the beam. (B) Sliced tissue with lesions formed at a plane parallel to the beam after sonications with different parameters with transducer having frequency 3.2 MHz at various focal depths. MR, magnetic resonance; PD, proton density

Movement of the transducer was also evaluated in an MRI scanner. MR thermometry maps were acquired during the sonication. Figure 13 shows the MR thermal map acquired during sonication at acoustical power of 57 W for sonication time of 10 s. A temperature increase of 55.8 C was recorded at the end of the sonication resulting in lesion formation. Figure 14A shows the PD MR image with fat suppression obtained on coronal plane. Figure 14B shows the sliced tissue with the lesions formed on a plane parallel to the ultrasonic beam. The average diameter of the lesions on the first row (focal depth 2 cm) was 7.47 mm with a corresponding average length of 12.9 mm. The average diameter of the lesions on the second row (focal depth 3 cm) was 9.9 mm and a respective average length of 27.1 mm.

### 3.4 In vivo evaluation of the FUS system

**FIGURE 15:**
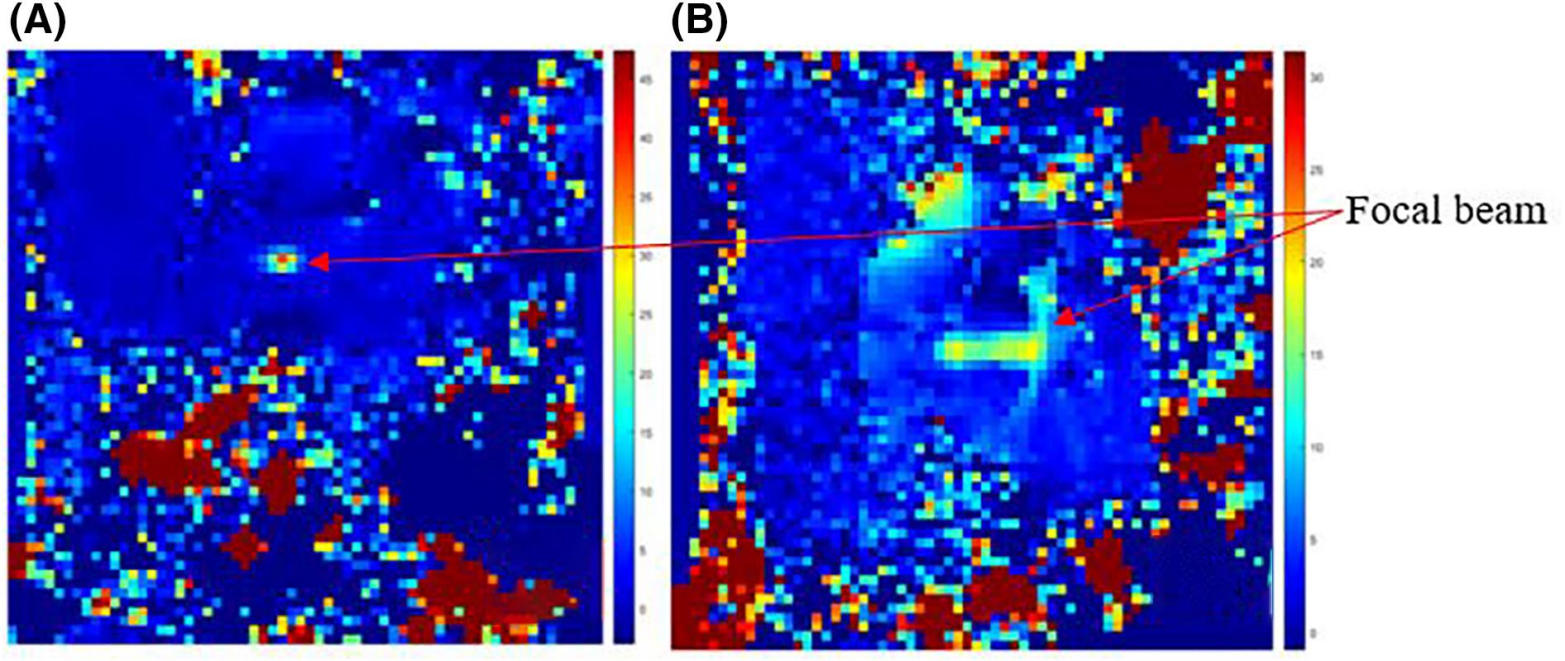
Temperature maps recorded using single‐shot EPI sequence after applying acoustical power 22.5 W for sonication time of 30 s at 2 cm focal depth, on in vivo rabbit thigh tissue with transducer having frequency 3.2 MHz. (A) Coronal plane. (B) Axial plane. EPI, Echo Planar Image

Sonication of the right thigh was executed at acoustical power of 22.5 W for a sonication time of 30 s at 2 cm focal depth. Figure 15A shows the MR temperature map acquired on coronal plane while Figure 15B shows the acquired map on axial plane. Temperature maps provided a near‐real time feedback of the temperature increase, with 39.3 C being recorded in the coronal plane.

**FIGURE 16:**
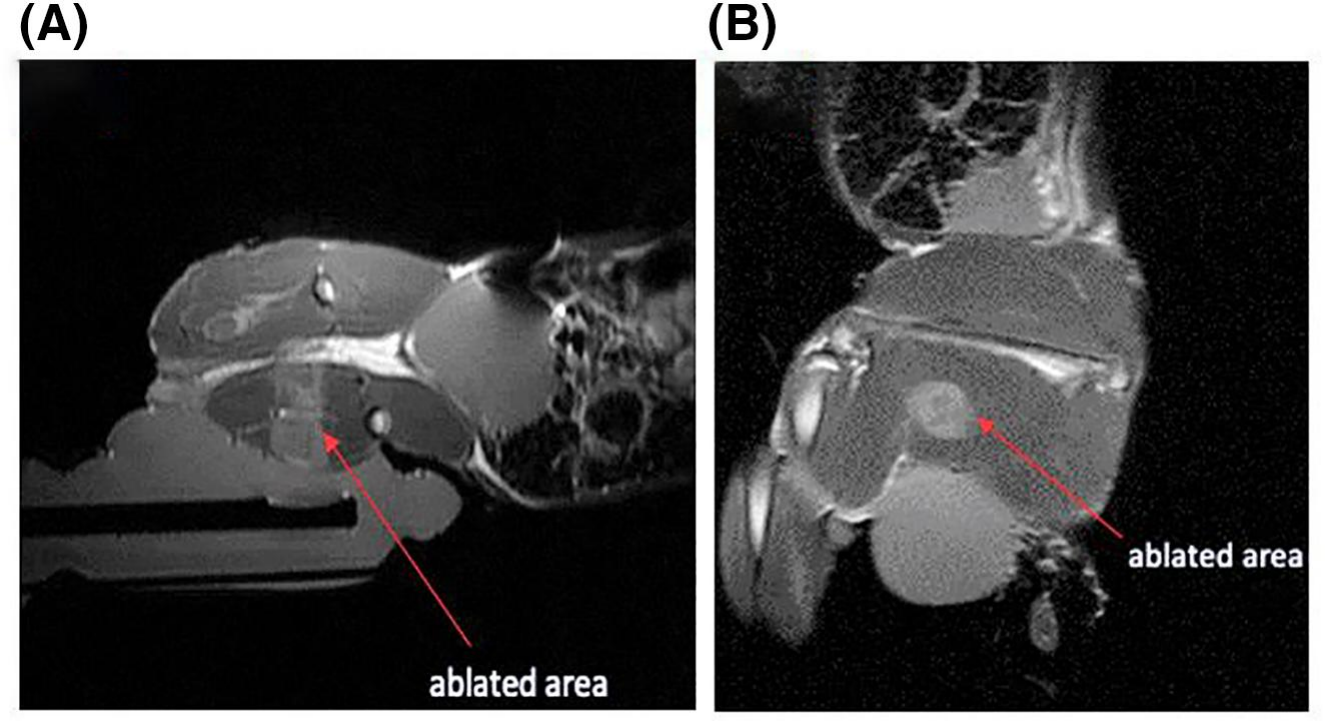
PD image with fat suppression obtained after applying acoustical power of 22.5 W for a sonication time of 30 s at 2 cm focal depth with transducer having frequency 3.2 MHz. (A) Sagittal plane, (B) Coronal plane. PD, proton density

**FIGURE 17:**
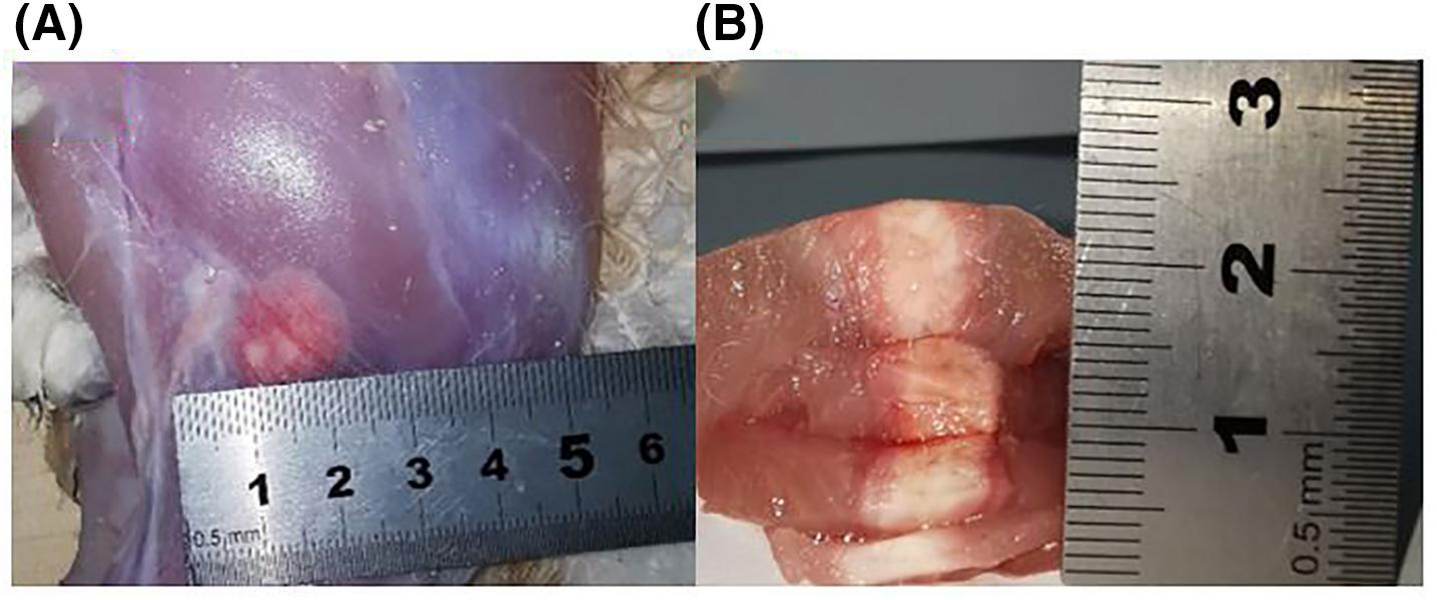
Lesion formed on in vivo rabbit thigh tissue resulting exposure at acoustical power of 22.5 W for sonication time of 30 s at 2 cm focal depth with transducer having frequency 3.2 MHz. (A) Lesions formed on plane perpendicular to the beam, (B) Lesions formed on plane parallel to the beam

The aforementioned temperature increase resulted in the formation of a thermal lesion. Figure 16 shows the acquired PD with fat suppression image of the ablated area. The lesion formed had a 16.4 mm diameter and 24.8 mm length as calculated from the MR image. Ante mortem dissection of the ablated area resulted in slightly different lesion dimensions. Figure 17A shows the lesion formed on a plane perpendicular to the beam while Figure 17B shows the lesion formed on a plane parallel to the beam. The formed lesion had a 15 mm diameter and 24 mm length as measured with the calliper.

## 4 DISCUSSION

A MRgFUS robotic device with a transrectal probe has been developed for localized therapy of PC. The proposed system has been an evolution of previous robotic prostate devices developed by some researchers from this group^37^, ^38^, ^39^ and has been inspired by existing FUS devices featuring an endorectal probe.^11^ ^,^ ^12^ ^,^ ^32^ The robotic device offers movement in 5 DOF and can be placed on the table of any commercial cylindrical MRI scanner up to 7 T.

The device has been designed to obtain minimal space in the MRI bore as to not discomfort the patient. Several improvements have been implemented since previously developed designs.^37^, ^38^, ^39^ The motion stages of the proposed device have been reduced in size to be included in an ABS enclosure thereby improving aesthetically the appearance of the device in the clinical setting. The design of the angular axis Θ was the most challenging considering the space limitations. The planetary mechanism was chosen since it was the most compact unit compared to equivalent mechanisms. The small and symmetrical size of the planetary mechanism offers an improved overall device appearance. Additionally, the inclusion of a manually controlled linear axis (Y) permits left to right movement on the base of the robotic device, allowing an easier insertion of the transrectal probe without requiring reposition of the patient. Moreover, the diameter of the probe was reduced compared to previous designs^37^, ^38^, ^39^ and therefore the pressure to the rectum is reduced. Furthermore, motion along the axis of the rectum (X) is achieved through two jackscrew mechanisms compared to the single mechanism previously used.^39^ Consequently, there is a uniform force application, resulting in a smoother and more accurate motion. Moreover, addition of the enclosure permits placement of the whole device in a sterilization chamber for disinfection between procedures. Taking into consideration the materials used, ethylene oxide sterilization is recommended to achieve the recommended sterility assurance level of 10^‐6^.^42^ The use of radiation is not recommended due to possible colour degradation nor is steam sterilisation due to deformation of the ABS enclosures attributable to the high temperatures used in autoclaves. In future designs, the transrectal probe can be designed to be detachable so as to allow for easier sterilization procedure.

The device was tested in a 1.5 T scanner for MR compatibility of the transducer and robotic system by measuring the SNR for a variety of activation conditions. The SNR was measured for T2‐W FRFSE, FSPGR and EPI sequences. Overall, the presence and activation of the transducer and the robotic device in the MR bore did not significantly affect the SNR. No impair effect on the MRI images as well as utilisation of non‐magnetic materials, such as ABS, for development ensured the MR compatibility of the device, hence providing the ability of obtaining MR thermometry maps during sonications for visualization of the thermal dose. Although EPI resulted in the largest SNR drop upon powering of the electronic system, it was the sequence utilised for the acquisition of MR thermometry maps since it results in faster acquisition times. Thereby, a better monitoring is attained due to the near‐real time nature of MR thermometry.

The ability of the transducer to induce high temperatures was evaluated on ex vivo tissue. However, the focus location of the transducer in ex vivo porcine tissue was shifted, resulting in a large increase of temperature and creation of lesion at the tissue interface. This temperature increase was probably due to reflection of the ultrasonic beam between the tissue interface and the degassed water. Moreover, sonications in a grid pattern resulted in lesions of constant diameter and slightly varied length, also formed on the interface. This along with their tadpole shape, indicates the effect of cavitational mechanisms during formation. Ex vivo tissue undergoes autolysis resulting in higher contents of air proportional to post‐mortem interval.^43^ The presence of air bubbles results in absorption of the propagated ultrasound energy, resulting in increased amounts of energies near the transducer, and thus the formation of lesions in the pre‐focal region. Being MR compatible, movement of the transducer was also evaluated in ex vivo tissue in the MRI environment. Lesion formation was dependent on focal depth; increased amounts of energy (power × time) needed for lesion formation at increased focal depth. Lesion diameter and length at the same focal depth increased with higher amounts of energy.

After indications that the FUS system was able to create lesions on excised tissue, its efficiency in ablating in vivo thigh tissue on rabbit model was assessed inside the MRI environment. The lesion was formed in situ ensuring that necrosis is achieved only on the targeted area, with no damages in intervening tissue. Monitoring of the vital signs of the rabbits was used for the designation of adverse effects. The rabbits remained in deep anaesthesia until euthanasia with mild level severities experienced during the experimental procedure. One limitation of this study was the size of the rabbit thigh which is around 20 mm thick. Normally, better focussing can be achieved with a 40 mm thickness with this type of transducer.

The MRI compatibility of the device and the ability of the transducer to provide repeatable lesions is making the proposed device an excellent candidate for the future use in clinical environments for the treatment of PC. The device will be superior from the existing ultrasound guided devices due to the MRI guidance and the ability of acquisition of thermal maps during treatment. Compared with the ExAblate system, the proposed device will be more cost effective since it uses a single element focused transducer. As a result, the design of this device is more compact and easier to handle during treatment. Compared with the TULSA‐PRO, the device offers a transrectal probe which provides transducer proximity to a larger area of the prostate gland and avoids urethral complications. Moreover, the TULSA‐PRO utilises unfocused elements, resulting in induced heating at intervening areas of the gland. The proposed device is also compatible with MRI scanners from all major manufacturers, making it superior from existing systems that are only compatible with specific manufacturers. Although this is still a preclinical prototype, the use of easily sourced materials for development (ABS), as well as the indicated fabrication method (3D printing) allow the system to be offered at a lower cost compared with others^44^ upon its introduction in the clinical environment.

Additionally, upon introduction of the device in the clinical environment, fiducial marks have to be placed on the transducer in order to allow registration of its home position relative to the MRI image. It is possible in the current prototype to visualize in the same image the transducer and the targeted anatomy. Therefore, since the radius of curvature of the ultrasonic transducer is known, it is possible to identify the focal spot during MR thermometry thus improving the treatment planning for this system. Additionally, during acquisition of high‐resolution images (lesion detection) both transducer and tissue of interest are both visible on the same image.

In a clinical setting, pre‐treatment, MR images will identify the tumour location. Therefore, based on this information, the physician will insert the probe accordingly. During the operation, the physician might manually correct this depth based on MR images. Most likely, due to the long range of the X axis, it is possible that targeting of the tumour is achieved without any manual correction. In case the patient moves during a future clinical trial, a sensor might be needed to interrupt the treatment. A similar approach is applied in radiotherapy. The proposed device was designed according to the clinical standards established by other U.S.‐guided devices already deployed in the clinical setting. These include accuracy of the robotic system, efficacy and safety of the ultrasonic transducer, MR compatibility, integration with an imaging modality and ergonomics.

This device can be utilized in the clinical environment after extensive preclinical experiments involving animals for the functionality of the device in prostatic tissue. The in vivo ablations on rabbit muscle confirmed that the device is capable of inducing in situ tissue necrosis while the procedure does not compromise animal welfare and wellbeing. Although its efficacy in ablating prostatic tissue was not performed due to limitations on animal size and anatomy, the experiments conclude that the device offers promising results to enter the market of PC FUS treatments. With minor changes to the transducer element, the device can be used endovaginally for the treatment of fibroids or endovaginal tumours. The main disadvantage of this device compared to U.S.‐guided devices is the additional cost of utilizing an MRI scanner during treatment. However, for life‐threatening tumours encountered in the prostate gland, this disadvantage can be overlooked due to the increased benefit offered by MR thermometry during treatment.

## 5 CONCLUSION

The development and evaluation of an MRI compatible robotic system featuring motion in 5 DOF and a single‐element transducer intended for HIFU therapy of PC was presented. Evaluation of the robotic device included MRI compatibility in terms of SNR decrease as well as the ability of the ultrasonic transducer to induce high temperatures and form lesions on ex vivo pork loin tissue and in vivo rabbit thigh tissue. The slight reduction in SNR and the use of non‐ferromagnetic materials ensured the MRI compatibility of the system. Moreover, the transducer has been proven successful in forming lesions on ex vivo and in vivo tissues. Although the ability of the transducer in causing irreversible necrosis on prostatic tissue has not been proven, the preliminary sensed data suggest a possible future use of the robotic device in the clinical environments for localised therapy of PC. Future studies could entail in vivo animal experiments for assessing the efficiency in prostatic tissue ablation as well as advancement of the preclinical device so as to achieve clinical requirements for possible introduction in the market.

## CONFLICT OF INTERESTS

The authors declare no conflict of interests.

## AUTHOR CONTRIBUTION

Marinos Giannakou contributed in the development of the robotic device and its evaluation. Theocharis Drakos contributed in the evaluation of the device in ex vivo and in vivo experiments. George Menikou contributed in the MRI experiments. Nikolas Evripidou contributed in the development of the electronic system and evaluation experiments. Antria Filippou contributed in the evaluation of the device in ex vivo experiments and drafting of the manuscript. Kyriakos Spanoudes contributed in the in vivo evaluation of the device. Leonidas Ioannou contributed in the MRI experiments. Christakis Damianou supervised the development and evaluation of the device and drafting of the manuscript.

### DATA AVAILABILITY STATEMENT

The data that support the findings of this study are available on request from the corresponding author. The data are not publicly available due to privacy or ethical restrictions
